# Correlations between family history and cancer characteristics in 2256 breast cancer patients

**DOI:** 10.1038/sj.bjc.6601905

**Published:** 2004-05-25

**Authors:** A Molino, M Giovannini, R Pedersini, M Frisinghelli, R Micciolo, M Mandarà, M Pavarana, G L Cetto

**Affiliations:** 1 Department of Medical Oncology, University of Verona, Verona, Italy; 2 Department of Statistics, University of Trento, Italy

**Keywords:** breast cancer, family history, prognostic factors

## Abstract

A comparison of 692 early invasive breast cancer with, and 1564 without, a family history of breast cancer showed that the former were younger at diagnosis (*P*=0.002), had smaller tumours (*P*=0.012), were more frequently oestrogen receptor positive (*P*=0.006) and diagnosed preclinically (*P*<0.001).

A long recognised risk factor for breast cancer is a family history of the disease, although the majority of affected women do not have an affected close relative, and only 5–10% do have a true hereditary predisposition (Carter, 2001). The overall risk of developing breast cancer is 1.9–3.9 times higher in women with an affected mother or sister (Collaborative Group on Hormonal Factors in Breast Cancer, 2001), but only a few studies have investigated the characteristics of breast cancer in women with a family history. We analysed the pathological, biological and clinical features of breast cancer in patients with (FH+) and without (FH−) a family history of breast cancer, the former being further subdivided into those with an affected first- or second-degree relative.

## MATERIALS AND METHODS

A total of 2256 women with early invasive operable breast cancer, who underwent surgery at Verona Hospitals between January 1992 and April 2001, were asked at their first visit whether they had any first- or second-degree relatives who had had breast cancer. Our analysis first compared those reporting at least one affected relative (FH+) with those reporting no affected relative (FH−); subsequently, the FH+ patients were divided into those with at least one first-degree relative (1st DFH) and those with only second-degree relatives with breast cancer (2nd DFH): only first- and second-degree relatives were considered in order to reduce ascertainment bias. Answers were always checked at the subsequent visit (at the time of the first cycle for the patients receiving chemotherapy, after 3 months for the others).

All of the patients were assigned a UICC pathological TNM stage. On the basis of pathologist-defined tumour size, patients were divided into three categories: pT1 (<2 cm), pT2 (2–5 cm) and pT3 (>5 cm); the number of pathologically positive axillary nodes was divided into none, <3, 4–10 and >10; tumour grading was recorded as G1 (well differentiated), G2 (moderately differentiated) or G3 (undifferentiated).

Immunohistochemistry (IHC) defined oestrogen (ER) and progesterone receptor (PgR) status and was considered positive if more than 10% of the cells were stained for either. The replicative cell fraction was IHC stained using the Ki-67 monoclonal antibody (Mab-DAKO-PC); given the lack of an accepted cutoff point, the results were arbitrarily classified as low, medium or high (⩽10%, 11–25% or >25% of stained cells). C-*erb*B-2 levels were determined by IHC using the DAKO-PC monoclonal antibody, and considered positive if at least one cell was stained.

At their first visit to the Department of Medical Oncology, all patients were asked about their disease presentation and divided into those who underwent mammography and ultrasonography because of breast discomfort or a lump (symptomatic) and those without any subjective symptoms (asymptomatic). A public screening programme has been active in Verona since July 1999, before which 30% of our patients had undergone mammography and ultrasonography even in the absence of subjective symptoms.

For the analysis of the pathological, biological and clinical differences by age at diagnosis, the patients were arbitrarily divided into seven age groups (<35, 36–45, 46–55, 56–65, 66–75, 76–85 and >85 years).

The *χ*^2^ test was used to compare the prevalence of FH+ (1st and 2nd DFH) and FH− women in relation to all the variables.

As it is known that younger women have a higher risk associated with a family history, an age-adjusted analysis was made to compare the prevalence of FH+ women across the categories of the other considered variables.

Significance was tested using the likelihood ratio statistic and a significance level of 0.05.

## RESULTS

The overall prevalence of FH+ women was 30.7% (692), of whom, 356 (51.4%) were classified as 1st DFH and 336 (48.6%) as 2nd DFH. Table 1

**Table 1:** Correlation between breast cancer characteristics in patients with and without FH

				***P-*value**	***P-*value**
	**FH− no.^a^**	**FH+ (1st degree) no.^b^**	**FH+/total %**	**Raw**	**Adjusted^c^**
*pTumour size*				0.006	0.012
T1	1036	502 (262)	32.6		
T2	495	173 (85)	25.9		
T3	33	17 (9)	34.0		
					
*No. of positive nodes*				>0.5	>0.5
0	947	412 (205)	30.3		
1–3	360	159 (82)	30.6		
4–10	135	66 (31)	32.8		
>10	63	27 (14)	30.0		
					
*ER*				0.021	0.006
Negative	284	99 (48)	25.8		
Positive	1199	561 (288)	31.9		
					
*PgR*				0.211	0.264
Negative	511	222 (104)	30.3		
Positive	799	394 (206)	33.0		
					
*Proliferative index*				0.105	0.073
Ki-67=0–15%	828	400 (205)	32.6		
Ki-67=16–25%	311	140 (74)	31.0		
Ki-67=26–100%	276	101 (47)	26.8		
					
*Grading*				0.476	0.397
G1	165	79 (39)	32.4		
G2	691	320 (168)	31.7		
G3	369	150	28.9		
					
*C-erb B2 status*				0.107	0.065
Negative	427	239 (117)	35.9		
Positive	298	135 (69)	31.2		
					
*Age at diagnosis (years)*				0.002	—
⩽35	32	19 (6)	37.3		
35–44	190	84 (28)	30.7		
45–54	371	201 (107)	35.1		
55–64	376	184 (84)	32.9		
65–74	373	137 (80)	26.9		
75–84	185	51 (38)	21.6		
⩾85	37	16 (13)	30.2		
					
*Disease presentation*				<0.001	<0.001
Asymptomatic	502	302 (144)	37.6		
Symptomatic	1013	372 (198)	26.9		

ER=oestrogen receptors; PgR=progesteron receptors.

a No family history of breast cancer.

b Family history of breast cancer.

c *P*-value adjusted for age at diagnosis.

shows the crude and age-adjusted prevalence of FH+ women across the various categories. Family history was significantly associated with tumour size, ER, age at diagnosis and disease presentation. No significant association was found between family history and nodal involvement, PgR, Ki-67 status, grading or c-erb B2 levels (Table 1).

Associations with degree of familial relationship were investigated only for the variables significantly associated with a family history when a more general definition was used (tumour size, ER, age at diagnosis and disease presentation). Only age at diagnosis was significant (χ^2^=36.0; *P*<0.001): older women were more likely to have a first-degree relative (76.1% of those aged >74 years had an affected first-degree relative *vs* 51.9% of those aged 45–74 and 33.0% of those aged <45 years; data not shown).

## DISCUSSION

A positive family history is now an established risk factor for breast cancer, but few studies have focused on the characteristics of breast cancer in FH+ women. We have done so because physicians can rarely study genetic patterns in clinical practice, and must therefore rely on patient-supplied FH reports despite their possible inaccuracy (Slattery and Kerber, 1993; Eerola *et al*, 2000). Furthermore, some tumours will develop in relatives after the patient has been examined.

We have only considered first- and second-degree relatives, the percentages of which were similar to those reported in other studies (Collaborative Group on Hormonal Factors in Breast Cancer, 2001). Our adjusted results suggest significant differences in FH+ tumours, which seem to be smaller, more often ER+, and more likely to be diagnosed at a younger age and at an asymptomatic stage. The published data are few and inconsistent, and usually not subject to multivariate analysis adjusted for age; in fact, only Kreiger about receptor status (Kreiger *et al*, 1991) and Swede about c-erbB2 (Swede *et al*, 2001) compared FH with clinical and pathological characteristics in a multivariate analysis adjusted for age as in this paper. Most have not found any significant difference in tumour size between FH+ and FH− patients (Fukutomi *et al*, 1993; Israeli *et al*, 1994; Tsuchiya *et al*, 1998; Russo *et al*, 2002), whereas Mohammed observed a trend (Mohammed *et al*, 1998) and Colditz reported a higher percentage of T1 in women FH+ (60%) *vs* FH− (54%) (Colditz *et al*, 1993). There were no significant differences in nodal involvement between our FH− and FH+ tumours. Some studies (Fukutomi *et al*, 1993; Mohammed *et al*, 1998) reported that FH+ patients were more likely to have tumours with fewer nodal metastasis, but others (Ruder *et al*, 1988; Israeli *et al*, 1994; Tsuchiya *et al*, 1998; Russo *et al*, 2002) found no significant difference.

Our univariate and multivariate analyses showed that FH+ tumours are more likely to be ER+, but there were no statistically significant differences in PgR, Ki-67 or grading. Some studies found that FH+ tumours are more likely to be ER− and PgR− (Kreiger *et al*, 1991; Huang *et al*, 2000), have a higher proliferation rate (Marcus *et al*, 1994) and higher grading (Mohammed *et al*, 1998), but none of the others found any differences (Fukutomi *et al*, 1993; Israeli *et al*, 1994; Tsuchiya *et al*, 1998; Russo *et al*, 2002).

In agreement with others (Fukutomi *et al*, 1993; Swede *et al*, 2001), we did not find any differences in relation to the more recent c-erbB2 marker.

Our FH+ tumours were more frequently diagnosed in an asymptomatic phase than the FH− tumours. This is probably due to the greater sensitisation induced by having one or more affected relatives, which encourages asymptomatic women to undergo diagnostic investigations; our data are similar to those of others (Colditz *et al*, 1993; Murabito *et al*, 2001) who have observed that FH+ women undergo more mammographies.

Our FH+ patients were younger at diagnosis, which confirms some previous data (Israeli *et al*, 1994), but not others (McCredie *et al*, 1997; Tsuchiya *et al*, 1998; Russo *et al*, 2002).

In conclusion, FH+ seems to be associated with an asymptomatic diagnosis (and therefore smaller and ER+ tumours), possibly because of the more intensive use of mammography or differences in biological behaviour. FH+ patients are younger, although it is not clear as to whether this reflects a truly earlier disease onset or an earlier diagnosis due to the more intensive use of mammography.

## References

[bib1] Carter RF (2001) BRCA1, BRCA2 and breast cancer: a concise clinical review. Clin Invest Med 24(3): 147–157 11437066

[bib2] Colditz GA, Willett WC, Hunter DJ, Stampfer MJ, Manson JE, Hennekens CH, Rosner BA (1993) Family history, age, and risk of breast cancer. Prospective data from the Nurses' Health Study. JAMA 270(3): 338–343 8123079

[bib3] Collaborative Group on Hormonal Factors in Breast Cancer (2001) Familial breast cancer: collaborative reanalysis of individual data from 52 epidemiological studies including 58 209 women with breast cancer and 101 986 women without the disease. Lancet 358(9291): 1389–1399 11705483 10.1016/S0140-6736(01)06524-2

[bib4] Eerola H, Blomqvist C, Pukkala E, Pyrhonen S, Nevanlinna H (2000) Familial breast cancer in southern Finland: how prevalent are breast cancer families and can we trust the family history reported by patients? Eur J Cancer 36(9): 1143–1148 10854948 10.1016/s0959-8049(00)00093-9

[bib5] Fukutomi T, Kobayashi Y, Nanasawa T, Yamamoto H, Tsuda H (1993) A clinicopathological analysis of breast cancer in patients with a family history. Surg Today 23(10): 849–854 7905302 10.1007/BF00311360

[bib7] Huang WY, Newman B, Millikan RC, Schell MJ, Hulka BS, Moorman PG (2000) Hormone-related factors and risk of breast cancer in relation to estrogen receptor and progesterone receptor status. Am J Epidemiol 151(7): 703–714 10752798 10.1093/oxfordjournals.aje.a010265

[bib8] Israeli D, Tartter PI, Brower ST, Mizrachy B, Bratton J (1994) The significance of family history for patients with carcinoma of the breast. J Am Coll Surg 179(1): 29–32 8019721

[bib9] Kreiger N, King WD, Rosenberg L, Clarke EA, Palmer JR, Shapiro S (1991) Steroid receptor status and the epidemiology of breast cancer. Ann Epidemiol 1(6): 513–523 1669531 10.1016/1047-2797(91)90023-6

[bib10] Marcus JN, Watson P, Page DL, Lynch HT (1994) Pathology and heredity of breast cancer in younger women. J Natl Cancer Inst Monogr 16: 23–34 7999466

[bib11] McCredie M, Paul C, Skegg DC, Williams S (1997) Family history and risk of breast cancer in New Zealand. Int J Cancer 73(4): 503–507 9389563 10.1002/(sici)1097-0215(19971114)73:4<503::aid-ijc8>3.0.co;2-3

[bib12] Mohammed SN, Smith P, Hodgson SV, Fentiman IS, Miles DW, Barnes DM, Millis RR, Rubens R (1998) Family history and survival in premenopausal breast cancer. Br J Cancer 77(12): 2252–2256 9649141 10.1038/bjc.1998.374 PMC2150398

[bib13] Murabito JM, Evans JC, Larson MG, Kreger BE, Splansky GL, Freund KM, Moskowitz MA, Wilson PW (2001) Family breast cancer history and mammography: Framingham Offspring Study. Am J Epidemiol 154(10): 916–923 11700246 10.1093/aje/154.10.916

[bib14] Ruder AM, Moodie PF, Nelson NA, Choi NW (1988) Does family history of breast cancer improve survival among patients with breast cancer? Am J Obstet Gynecol 158(4): 963–968 3364506 10.1016/0002-9378(88)90103-2

[bib15] Russo A, Herd-Smith A, Gestri D, Bianchi S, Vezzosi V, Rosselli Del Turco M, Cardona G (2002) Does family history influence survival in breast cancer cases? Int J Cancer 99(3): 427–430 11992413 10.1002/ijc.10342

[bib16] Slattery ML, Kerber RA (1993) A comprehensive evaluation of family history and breast cancer risk. The Utah Population Database. JAMA 270(13): 1563–1568 8371466

[bib17] Swede H, Moysich KB, Freudenheim JL, Quirk JT, Muti PC, Hurd TC, Edge SB, Winston JS, Michalek AM (2001) Breast cancer risk factors and HER2 over-expression in tumors. Cancer Detect Prev 25(6): 511–519 12132871

[bib18] Tsuchiya A, Kanno M, Nomizu T, Hatakeyama Y, Kimijima I, Abe R (1998) Clinical characteristics of breast cancer patients with family history. Fukushima J Med Sci 44(1): 35–41 9775529

